# Globally distributed *Xyleborus* species reveal recurrent intercontinental dispersal in a landscape of ancient worldwide distributions

**DOI:** 10.1186/s12862-016-0610-7

**Published:** 2016-02-15

**Authors:** Jostein Gohli, Tina Selvarajah, Lawrence R. Kirkendall, Bjarte H. Jordal

**Affiliations:** Natural History Collections, University Museum of Bergen, University of Bergen, P.O. box 7800, 5020 Bergen, Norway; Department of Biology, University of Bergen, P.O. box 7800, 5020 Bergen, Norway

**Keywords:** Ancestral reconstruction, Anthropogenic effects, Bark and ambrosia beetles, Biological invasions, Dispersal, *Xyleborus*

## Abstract

**Background:**

Invasive species can have devastating effects on native ecosystems and therefore impose a significant threat to human welfare. The introduction rate of invasive species has accelerated dramatically in recent times due to human activity (anthropogenic effects), with a steadily growing pool of widespread tramp species. We present an in-depth analysis of four pantropical species of *Xyleborus* ambrosia beetles (*Xyleborus volvulus, Xyleborus perforans, Xyleborus ferrugineus,* and *Xyleborus affinis*) with similar ecology (fungus cultivation in dead wood), reproductive biology (permanent inbreeding) and genetic system (haplodiploidy). The unique combination of reproductive traits and broad host plant usage pre-adapts these beetles for colonizing of new areas.

**Results:**

We found that all four species were broadly distributed long before human-assisted dispersal became common, and that the impact of anthropogenic effects varied among the species. For *X. volvulus, X. perforans,* and *X. affinis* there was evidence of ancient establishment in numerous regions, but also of abundant recent introductions into previously colonized areas. For *X. ferrugineus*, we found clear biogeographical structuring of old clades, but little evidence for recent successful introductions.

**Conclusions:**

Our results indicate that current human-aided transoceanic dispersal has strongly affected the genetic makeup of three of the species in this study. However, current biogeographical patterns of all four species are equally, if not more strongly, influenced by ancient establishment on different continents.

**Electronic supplementary material:**

The online version of this article (doi:10.1186/s12862-016-0610-7) contains supplementary material, which is available to authorized users.

## Background

Invasive species constitute a major threat to native ecosystems worldwide [1, 2]. Many different types of organisms can be successful colonizers, and particularly those easily transported by humans over long distances [3]. Anthropogenic dispersal is therefore regarded as the most significant factor in explaining threats to ecosystems [2, 4] and agricultural crops [5]. However, long-range dispersal is not always human-aided and the relative importance of natural vs human-aided dispersal is often not obvious.

Even though many species are introduced to new continents or oceans every year, only a few become permanently established [6]. Serendipity plays a role in propagule establishment, but flexible habitat preferences and high propagule pressure are certainly important [7, 8]. Certain organisms have biological traits that likely facilitate establishment after dispersal [pre-adapted colonizers; 6, 9, 10]. Among invasive insects, mites, annelids, and plants, species that reproduce by sibling mating (or selfing) or by clonal reproduction through parthenogenesis are overrepresented [11–14]. For instance, the bark and ambrosia beetle fauna (Scolytinae) on remote oceanic islands is generally represented by two or three times more sib-mating species compared to the mainland [11], and nearly three-quarters of the established alien Scolytinae in North America are brother-sister maters [15].

With both inbreeding and parthenogenesis, a single mated female is sufficient for a species to become established in a new area [10, 16, 17]. Reproductive assurance is advantageous to insects such as bark beetles that depend on locating scattered, ephemeral resources, and enables long-distance colonization and establishment. For normally outbreeding species, a large number of introduced individuals [18] or repeated introductions [19] can ameliorate the effects of inbreeding depression. Permanent inbreeders [20], on the other hand, are expected to have purged strongly deleterious alleles early in a lineage's history, and both parthenogens and inbreeders have been under strong selection for combinations of genes which work well together. Regularly inbreeding species are thus expected to be largely immune to the effects of inbreeding that result from bottleneck events such as colonisations [21], and are therefore relatively unaffected by many ecological and genetic mechanisms normally causing Allee effects [22].

In both the North American and European wood boring fauna, exotic species are disproportionately ambrosia beetles that cultivate and feed upon mutualistic fungi in tunnels they excavate in dead trees [15, 23]. The fungal symbionts are host generalists, which enable the beetles to breed in many host plants families [24, 25], pre-adapting them for successful colonization of distant regions [4, 10–13]. Further, in contrast to the native faunas, exotic ambrosia beetles are almost all inbreeders [26, 27].

The ambrosia beetle genus *Xyleborus* contains many abundant and widespread invasive species [28–32]. All inbreed by sibling mating and all are haplodiploid (males are haploid and produced by unfertilized eggs). By mating with siblings,having the potential for re-mating with clonally produced sons in the absence of mates, and the ability to grow fungi for food in almost any kind of wood, *Xyleborus* and similar ambrosia beetles are exceptionally efficient at colonizing and establishing in new areas.

Human activity results in a constant stream of introductions of bark and ambrosia beetles to new areas [26, 33–36]. For wood boring beetles such as these, the most common mechanism for intercontinental dispersal is transport in timber and wooden packing materials [26, 34]. They survive such transport particularly well, being ensconced in material which provides food, a buffered microenvironment, and protection from most natural enemies. Hence, a wide variety of exotic bark and ambrosia beetle species are regularly trapped near harbours around the world [26, 27, 34, 37–39]. While anthropogenic effects strongly influence the recent spread of these beetles, many species of *Xyleborus* are also known from the earliest examinations of tropical scolytine fauna on different continents [40–42]. Their presence over several centuries may well be due to anthropogenic factors, but could also indicate prehistorical dispersal. It is therefore uncertain if human transport alone can explain the pantropical distribution of these beetles.

In order to test the relative importance of ancient vs. modern dispersal for current geographical distributions, we reconstructed the biogeographical history of four *Xyleborus* species. The species were chosen because they are largely pantropical in distribution and are among the most numerous ambrosia beetles wherever they are found [30, 43–45]. Due to their nearly global distribution, it has not been possible to determine their geographical origin. We envision three alternative scenarios for the biogeographical history of these species: (i) If *Xyleborus* beetles became widely established before the dawn of human influence on species distributions, we expect evidence for ancient establishment in multiple areas. Ancient distributions would be reflected in multiple divergent clades that are largely restricted to specific regions. High haplotypic diversity within several geographical regions would furthermore imply stable populations since prehistorical time. (ii) Contrarily, if a current pantropical distribution is due largely to multiple human-aided dispersal events, we expect the source area to be represented by a single genetically diverse clade. Very recent colonisations of new regions would then be evident from young derived clades nested within the aforementioned clade. (iii) A third possible result is a combination of the features from (i) to (ii), which would result from ancient dispersal to multiple regions followed by recent human-aided introductions to the same and other areas.

## Methods

Females of the four focal species were sampled from four continents to 20 countries (Additional file 1: Table S1). Beetles were collected by hand, or in ethanol-baited flight intercept traps, or light traps, and preserved in 95 % ethanol. All specimens were identified based on morphology (BHJ and LRK) before extraction. We extracted DNA from 53 *Xyleborus ferrugineus*, 62 *X. affinis*, 25 *X. volvulus,* and 30 *X. perforans* individuals. We included sequences from *Coccotrypes cyperi*, *C. advena*, *Ozopemon brownei*, and *Xylosandrus morigerus* as outgroups [46], and we included data from nine related *Xyleborus* species to test for monophyly in our focal species (Genbank acc. no and metadata in Additional file 1: Table S3).

We sequenced one mitochondrial gene, cytochrome oxidase I (COI), and one nuclear gene, Elongation Factor 1-alpha (EF1α). The rapidly evolving mitochondrial gene is suited for examining population structure, while the nuclear gene may be more informative when attempting to resolve older relationships among species. A subset of individuals representing all identified COI haplo-groups was selected for EF1α sequencing (Additional file 1: Table S1). Before combining the two loci in an analytical framework, we assessed their phylogenetic concordance with a Congruence Among Distance Matrices (CADM) test [47]. Using the function tanglegram from the R package dendextend, we visualized separate phylogenetic reconstructions for each locus—and their concordance—as a tanglegram.

We extracted genomic DNA using E.Z.N.A® Tissue DNA Kit (Omega Bio-Tek, Atlanta, GA, USA) following the manufacturer’s protocol. 610 base pairs (bp) of COI gene and 1043 bp of the EF1α nuclear gene were amplified and sequenced using primers from Normark et al. [48; Additional file 1: Table S2]. PCR reactions were performed in 25 μL volume with 10 μM of each forward and reverse primer, 0.25 mM dNTPS, 0.625 U HotStar *Taq* DNA Polymerase, 17.4 μL ddH_2,_0, 2.5 μL of 10x PCR buffer, 25 mM MgCl_2_ and 1.0 μL of DNA template using the following PCR cycle: 95°, 15 min; 35 cycles [94°, 30s; 48°, 45 s; 72°, 60s]; 72°, 7 min. PCR reactions for EF1α were performed in 26 μL volume containing similar ingredients and concentrations as for CO1 gene amplifications, but with 2.0 μL of DNA. A touchdown PCR profile was employed for this nuclear gene: 95°, 15 min; 13 cycles TD[94°, 30s; 58–44°, 45 s; 72°, 30s]; 26 cycles [94°, 30s; 44°, 45 s; 72°, 30s]; 72°, 6 min. We purified amplified DNA using ExoSAP (Exonuclease I—Shrimp Alkaline Phosphatase) and sequenced in both directions using standard protocol for ABI BigDye® Terminator v3.1 Cycle sequencing kit (Applied Biosystems).

Sequence contigs were assembled and edited using SEQMAN II, a contig assembly module from DNASTAR Lasergene6®, and aligned using ClustalW [49] in BioEdit 7.0.1 [50]. The alignments were further refined using amino acid translation. We found no indications of nuclear insertions (NUMTs) of the COI gene in our sequenced data (all single-peak chromatograms, no stop codons or indels). COI (acc.no: KP941137-KP941327) and EF1α (acc.no: KP941328-KP941418) sequences are deposited in Genbank.

The phylogenetic and biogeographical reconstruction included both genes and all sequence data (194 individuals: the four focal species, outgroup, and ingroup), and seven geographical regions (Afrotropical mainland, Afrotropical islands (Indian Ocean), Neotropical mainland, Neotropical islands (Pacific Ocean), Indo-Malaysia, Australasia, Australasian small islands (Pacific Ocean). Haplotype networks were constructed independently for both COI and EF1α for this dataset using haploNet from the pegas R package [51]. 2) Ancestral reconstruction of geographical distribution was done by defining geographical location as discrete traits in BEAST, using a symmetric trait substitution model. Social network was inferred with BSSVS [52] in BEAST. The biogeographical reconstruction was performed using a conditional reference prior [53]. Since we were interested in identifying supported diffusions (dispersal/migration routes) for each species, we performed analyses on each individual species set using the same approach as outlined above. The results from these latter analyses were further analysed in SPREAD [54] (a tool developed for epidemiology studies, which can also be used to visualize biogeographical histories in general, e.g. [55, 56]). A Bayes factor cutoff of 10 was implemented so that only highly supported diffusions were returned by the software.

The best-fit evolutionary models of sequence evolution were determined with jModeltest 2.1.3 [57, 58] (Additional file 1: Table S4 and Additional file 1: Table S5), and phylogenetic and biogeographical reconstructions were performed with BEAST v1.8.2 [59]. The Yule speciation process tree prior was used in the reconstruction which included all species and the constant size coalescence tree prior was used for reconstructions of individual species datasets. The larger dataset was run for 70 M and the smaller datasets for 20 M generations. Mean values and effective sample size (ESS values) for all parameters were obtained using Tracer [60] with 10 % burn-in (Additional file 1: Table S4 and Additional file 1: Table S5). Maximum clade credibility trees were obtained using TreeAnnotator from the BEAST package.

In addition to phylogenetic and biogeographic analyses, we performed a molecular dating analysis using COI and EF1α data. For time calibration, we referred to an unpublished phylogenetic analysis of Scolytinae (317 spp.) which include four fossil calibration points. The sister group of *Xyleborus* in this large analysis was used as the outgroup in the calibration analysis (Additional file 1: Table S3). In the original higher level analysis, *Xyleborus* was represented by *X. alluaudi* and *X. affinis*. We thus included *X. alluadi* in order to evaluate the age estimates for the MRCA for these two species in both analyses (as a qualitative test). The stem age of *Xyleborus* was set to 17.3 Ma based on our global Scolytinae dating, with a normal distribution prior (stdev=0.173 myr). Since our system is relatively young and contain similar species, we opted for a strict clock rate prior with rate estimation for both markers and a Yule speciation prior in BEAST.

Tests of molecular variance (AMOVA) were performed in Arlequin v3.5 [61]. We performed two AMOVA tests based on different sets of geographical regions. In the first test, we clustered populations into the same seven regions as used in the large phylogenetic reconstruction (Afrotropical mainland, Afrotropical islands (Indian Ocean), Neotropical mainland, Neotropical islands (Pacific Ocean), Indo-Malaysia, Australasia, Australasian small islands (Pacific Ocean)); in the second test, we merged the oceanic regions with their continental counterparts giving a total of four regions. COI and EF1α were analysed separately. We also estimated genetic diversity in terms of segregating sites, nucleotide and haplotype diversity (*S*, *π* and *H*) along with Tajima's *D*, which tests for neutrality and recent population expansion or contraction, using DNAsp [62]. The association between genetic and geographical distance was evaluated with mantel tests performed in *R* using mantel.rtest [63]. To evaluate the distribution of genetic distances among haplotypes within species, mismatch distribution plots for COI were created in *R* [64] using the function MMD from the pegas package [51].

## Results

**Fig. 1:**
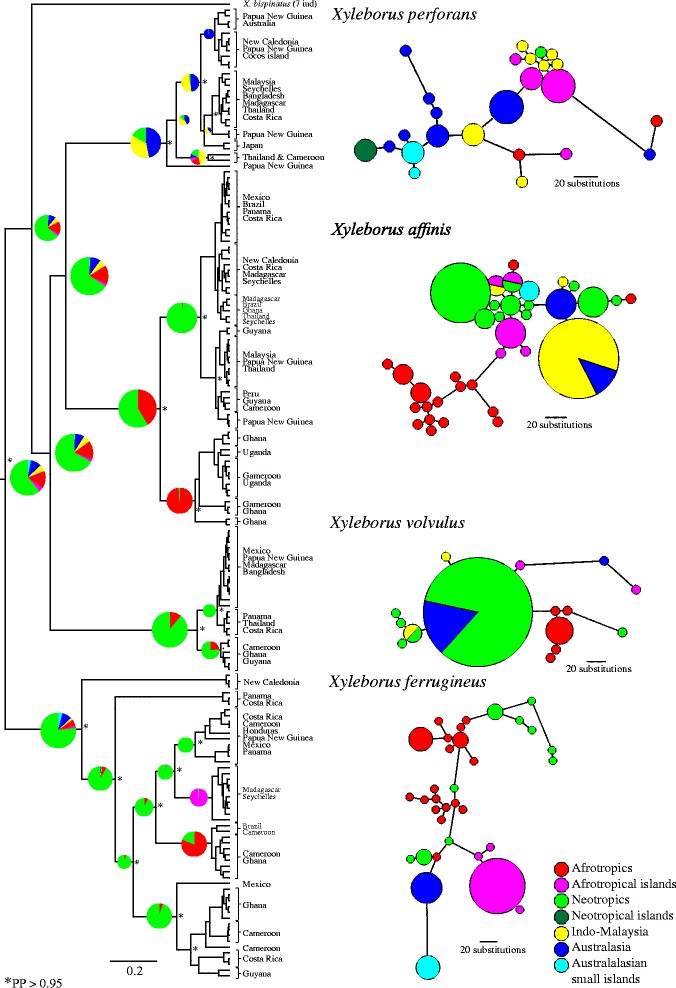
Biogeographical and phylogenetic reconstruction, and haplotype network. Legend: A phylogenetic tree for the four monophyletic focal species and one ingroup species (*X. bispinatus*) constructed using COI and EF1α. The phylogeny was constructed with sequences from 12 species as outgroup (Additional file 1: Table S3), which were removed from this figure. Posterior probability values are indicated by asterisks on nodes (*PP >0.95); numerous less important, yet strongly supported nodes (i.e., nodes nested deep within clades; PP >0.95), are not presented. Scale bars indicate branch length, which correspond to number of substitutions. Pie charts on branches show the relative probabilities of different geographical regions from the ancestral reconstruction. Circle sizes on trees contain no information, whereas circle sizes in the haplotype networks indicate number of individuals per haplotype. The haplotype networks shown here are based on COI only. Colour codes indicate regions, as shown in the bottom right corner, and are the same for pie charts in branches and in networks. Scale bars in the haplotype networks show the relationship between number of substitutions and branch lengths (these differ slightly among the haplotype networks)

A CADM global test of COI and EF1α was significant (Friedman's *X*^2^=3518.3; Kendall's *W*=0.820, *P*=0.0001), implying that the two molecular markers are congruent [47] and thus suitable for concatenation in an analytical framework. COI and EF1α phylogenies and their concordance are shown in a tanglegram (Additional file 1: Figure S2). Phylogenetic reconstructions indicated that all four focal species constitute monophyletic groups (Fig. 1).

Each species contained distinct intraspecific clades separated by a large number of substitutions, especially in the COI data (Fig. 1). Considerable geographical mixing was observed within subclades in three of the four species (*X. perforans*, *X affinis* and *X. volvulus*) except for one *X. affinis* clade that was purely Afrotropical. *Xyleborus ferrugineus* differed from the other species, containing numerous old clades that were generally restricted to distinct geographical regions. The differences noted between X*. ferrugineus* and the other three species were also apparent in the COI mismatch distribution plots (Additional file 1: Figure S3) showing large genetic distances between haplotypes in *X. ferrugineus*, while shorter genetic distances predominate in the mismatch distributions for the other three species.

The biogeographical reconstruction revealed that *X. ferrugineus* and *X. volvulus* most likely originated in the Neotropics (pie charts on phylogeny in Fig. 1). Neotropical and Afrotropical origin were both highly likely for *X. affinis*. For *X. perforans*, an Australasian origin had the highest probability, but Neotropical and Indo-Malaysian origins also had substantial probability scores.

COI haplotype networks were largely congruent with the phylogenies (Fig. 1). We found large genetic distances among clusters in all four species, indicating ancient splits between intra-specific lineages. Haplotype clusters were not geographically homogeneous for *X. affinis*, *X. volvulus* and *X. perforans*; clusters contained individuals predominantly from one geographical region together with one or a few individuals from a separate region. A few single haplotypes were shared by individuals from different geographical regions. Sequences of *X. ferrugineus* showed stronger geographical structuring, with geographically homogeneous haplotype clusters. We observed only one example of a very recent dispersal event (between the Neotropics and Afrotropics) in this species. There was also a tendency towards higher haplotypic diversity within geographical regions in *X. ferrugineus*, in particular the Afrotropical and Neotropical regions. The EF1α haplotype networks revealed little additional information to those of COI, but rather supported the general patterns seen in Fig. 1 (Additional file 1: Figure S4). As expected, there was significantly less fine scale resolution in the EF1α haplotype network compared to the COI network, which is due to the lower level of genetic variation in EF1α (Table 2).

**Fig. 2:**
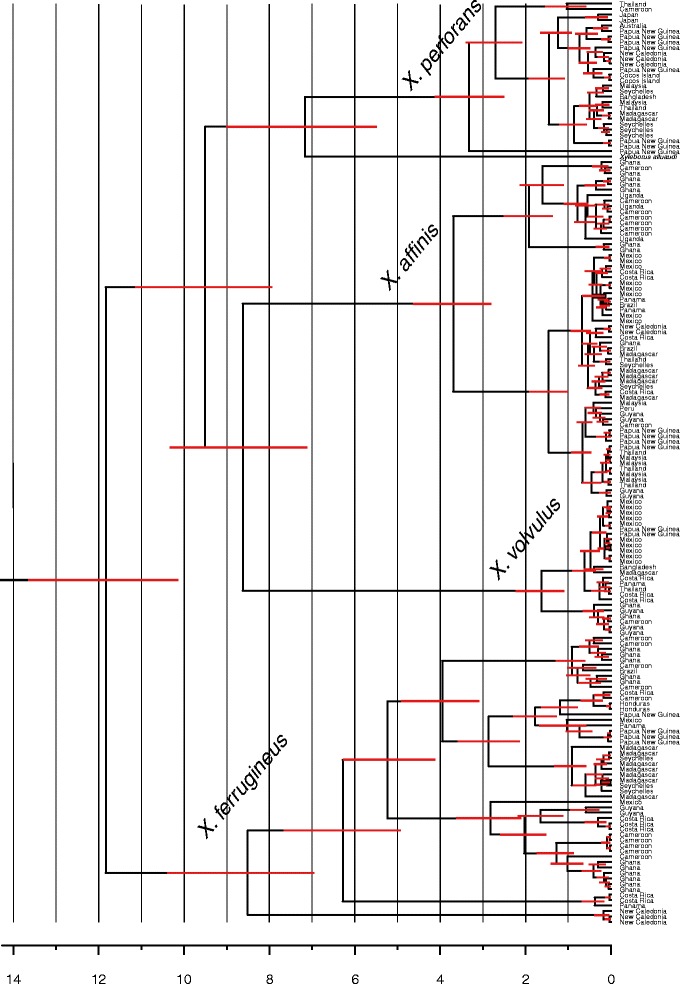
Dating analysis. Legend: Time calibrated analysis of five species of *Xyleborus* (*Xyleborus* stem=17.3 Ma). The scale axis is in million years. 95 % node height highest posterior density intervals are plotted on each node. Outgroup taxa are not shown (see methods)

The molecular dating analysis (Fig. 2) indicated a *Xyleborus* crown age of 11.8 Ma, with a 95 % highest posterior density (HPD) interval of [10.1–13.7 Ma]. The estimated minimum (crown) ages of our focal species were as follows: *X. perforans*-3.3 Ma; 95 % HPD [2.5–4.1 Ma], *X. affinis*-3.7 Ma; 95 % HPD [2.8–4.6 Ma], *X. volvulus*-1.6 Ma; 95 % HPD [1.1–2.2 Ma], and *X. ferrugineus*-8.5 Ma; 95 % HPD [6.9–10.4 Ma]. The estimated age of the *X. alluaudi*-*affinis* MRCA in our analysis (9.5 Ma), was quite close to the estimated age of the same node (10.8 Ma) in the larger analysis, from where we obtained our stem calibration age for *Xyleborus.*

In tests of molecular variance (AMOVA; Table 1), partitioning of variance varied significantly based on number of regions included (four vs. seven, treating oceanic islands as separate from continents). For all four species, there was a much larger proportion of variance among regions if oceanic regions were included compared to that observed when defining only four main regions for the analysis.

**Table 1:** Tests of molecular variance (AMOVA) for the four species and both genetic markers. The seven regions are Afrotropics, Afrotropical islands (Indian Ocean), Neotropics, Neotropical islands (Pacific Ocean), Indo-Malaysia, Australasia, Australasia small islands (Pacific Ocean). In the four regions the oceanic regions were merged with their continental counterparts

		Seven regions						Four regions					
	Source of variation	df	Sum of squares	Variance components	% variation	Fixation indices		df	Sum of squares	Variance components	% variation	Fixation indices	
*X. affinis:*													
	Among regions	5	446.7	8.5	59.6	Ф_CT_ : 0.59	**	3	280.3	4.7	34.0	Ф_CT_ : 0.34	*
COI	Among populations within regions	9	92.9	1.6	11.5	Ф_SC_ : 0.28	***	11	259.3	5.1	36.5	Ф_SC_ : 0.55	**
	Within populations	46	189.9	4.1	29.0	Ф_ST_ : 0.71	***	46	189.9	4,1	29.5	Ф_ST_ : 0.71	**
	Among regions	5	12.5	0.5	59.4	Ф_CT_ : 0.59	*	3	6.8	0.1	17.5	Ф_CT_ : 0.18	***
EF1α	Among populations within regions	5	2.5	0.1	14.0	Ф_SC_ : 0.34	*	7	8.2	0.4	54.1	Ф_SC_ : 0.66	**
	Within populations	19	4.0	0.2	26.6	Ф_ST_ : 0.73	***	19	4.0	0.2	28.5	Ф_ST_ : 0.71	*
*X. ferrugineus:*													
	Among regions	4	513.6	11.8	45.8	Ф_CT_ : 0.45	***	2	178.2	2.5	10.0	Ф_CT_ : 0.10	
COI	Among populations within regions	7	138.3	2.4	9.1	Ф_SC_ : 0.17		9	473.7	10.6	42.9	Ф_SC_ : 0.48	***
	Within populations	38	442.1	11.6	45.1	Ф_ST_ : 0.55	***	38	442.1	11.6	47.2	Ф_ST_ : 0.53	***
	Among regions	5	20.7	−1.0	−33.3	Ф_CT_ :−0.33		3	15.1	0.0	−1.5	Ф_CT_ :−0.01	
EF1α	Among populations within regions	3	12.2	2.2	77.0	Ф_SC_ : 0.58		5	17.7	1.3	45.8	Ф_SC_ : 0.45	
	Within populations	10	16.1	1.6	56.2	Ф_ST_ : 0.44	*	10	16.1	1.6	55.7	Ф_ST_ : 0.44	
*X. perforans:*													
	Among regions	6	182.5	1.3	9.3	Ф_CT_ : 0.09		3	88.6	−0.2	−1.5	Ф_CT_ :−0.01	
COI	Among populations within regions	7	128.5	6.3	44.3	Ф_SC_ : 0.49		10	222.4	7.7	54.6	Ф_SC_ : 0.54	***
	Within populations	20	132.6	6.6	46.4	Ф_ST_ : 0.54	***	20	132.6	6.6	46.9	Ф_ST_ : 0.53	***
	Among regions	5	6.4	−1.5	−98.0	Ф_CT_ :−0.98		3	4.1	−0.2	−12.9	Ф_CT_ :−0.12	
EF1α	Among populations within regions	1	3.0	1.5	99.0	Ф_SC_ : 0.50		3	5.3	0.2	14.9	Ф_SC_ : 0.13	
	Within populations	2	3.0	1.5	99.0	Ф_ST_ : 0.01		2	3.0	1.5	98.1	Ф_ST_ : 0.02	
*X. volvulus:*													
	Among regions	4	100.1	2.6	20.5	Ф_CT_ : 0.21		3	58.7	0.1	0.4	Ф_CT_ : 0.01	**
COI	Among populations within regions	5	71.8	2.5	19.2	Ф_SC_ : 0.24	**	6	113.3	4.3	35.5	Ф_SC_ : 0.36	
	Within populations	19	146.6	7.7	60.2	Ф_ST_ : 0.40	*	19	146.6	7.7	64.1	Ф_ST_ : 0.36	*
	Among regions							3	2.9	0.1	24.3	Ф_CT_ : 0.24	
EF1α	Among populations within regions			–				2	2.0	0.4	59.7	Ф_SC_ : 0.79	*
	Within populations							7	0.7	0.1	16.1	Ф_ST_ : 0.84	***

Fixation indices: among regions, φ_CT_; among populations within regions, φ_SC_; within populations, φ_ST_

*P*-values for % variation and corresponding fixation indices: *P < 0.05, **P < 0.01, ***P < 0.001

The test for seven regions for *X. volvulus* and EF1α was not possible since the oceanic regions were not represented in the data

*Xyleborus ferrugineus* was more genetically diverse, at both loci, than the other three species (Table 2). Tajima’s *D* was significant only for EF1α data, in *X. perforans* and *X. affinis*. Tajima’s *D* values for the two species (−1.901 and−1.848, respectively) indicate lower than expected genetic diversity. Mantel tests of COI genetic and geographic distance were significant and positive for three of the species (*P* <0.05; *X. volvulus: P*=0.077; Additional file 1: Figure S5); the strongest associations were identified in *X. perforans* (obs=0.326, *P*=0.011) and *X. ferrugineus* (obs=0.47, *P* <0.001). A Mantel test of EF1α genetic distance and geographic distance was significant and positive for *X. affinis* (obs=0.221, *P*=0.012) and *X. ferrugineus* (obs=0.412, *P*=0.021; Additional file 1: Figure S5). The same tests for EF1α were not significant for *X. volvulus* and *X. perforans*, which may be due to insufficient data.

**Table 2:** Three measures of genetic diversity and Tajima’s D for the four focal species

	loci	n	Number of segregating sites (S)	Total number of mutations	Nucleotide diversity (π)	Standard deviation of π	Total number of haplotypes	Haplotype diversity(Hd)	Standard deviation of Hd	Tajima's D
*X. volvulus*	COI	29	122	150	0.0457	0.0130	15	0.828	0.005	−1.573^*P>*0.10^
*X. perforans*	COI	34	119	156	0.0517	0.0097	24	0.979	0.012	−1.276^*P>*0.10^
*X. affinis*	COI	61	73	81	0.0424	0.0034	33	0.948	0.017	0.572^*P>*0.10^
*X. ferrugineus*	COI	50	134	169	0.0935	0.0040	33	0.968	0.000	0.502^*P>*0.10^
*X. volvulus*	EF1α	13	3	3	0.00125	0.0004	3	0.500	0.136	−0.143^*P>*0.10^
*X. perforans*	EF1α	9	14	14	0.00492	0.0021	4	0.583	0.034	−1.901^*P<*0.05^
*X. affinis*	EF1α	30	12	12	0.00141	0.0004	8	0.694	0.059	−1.848^*P<*0.05^
*X. ferrugineus*	EF1α	19	25	26	0.00634	0.0017	12	0.918	0.047	−1.056^*P>*0.10^

## Discussion

For all four *Xyleborus* species, we found conclusive evidence for colonization of separate continents millions of years ago (see Fig. 2). With the exception of *X. ferrugineus*, these species also show evidence of accelerated rates of colonization in recent times (Figs. 1 and 2), which likely stems from increased introductions through anthropogenic effects. However, the geographically broad distribution of these species, which was discovered during the earliest insect inventories in tropical Africa, South America and SE-Asia [40–42], is not primarily a result of introductions just prior to these first inventories. The results from our dating analysis and biogeographically structured COI haplotype clusters clearly indicate ancient dispersal and demonstrates that anthropogenic effects are not a necessity for the global spread of these or similar species.

The emerging picture of a clade with many widespread species dispersing since the time of their origin is a relatively novel finding and distinguishes this group from many other invasive organisms such as certain termites [65] and ants [66, 67] and several other bark and ambrosia beetles. The coffee berry borer (*Hypothenemus hampei*), for instance, has considerable genetic variation only in its native African region, with recent dispersal across continents following the cultivation of coffee [68]. Termites are particularly comparable in terms of ecology and includes the dry-wood inhabiting termites; despite dozens of widespread tramp species, none of these are ancient globetrotters, which is surprising given a flexible reproductive system and capacity for rafting in draft wood [65]. In this regard, termites show many similarities with a group of ambrosia beetles in the weevil subfamily Platypodinae, where a handful of recent world expansions is the exception among otherwise highly endemic beetles [69]. In this perspective it is truly remarkable that several species of *Xyleborus* have such a genuinely wide distribution pattern. However, they do possess a truly remarkable mode of reproduction that possibly holds the key for understanding their wide distribution. They are permanent inbreeders, hence inseminated before leaving their nest, and can even mate with sons produced from unfertilized haploid eggs [70]. Although there are many other species of Xyleborini that have very narrow distributions, species from this tribe are, together with another group of permanent inbreeders in the genus *Hypothenemus*, clearly overrepresented among the most widespread forest insects, indicative of their colonisation ability [70, 71].

Inferring the geographical origin of widespread *Xyleborus* is not straightforward given their complex patterns of repeated dispersal across and between continents. A close evolutionary relationship between a large clade of endemic Neotropical genera (*Sampsonius, Dryocoetoides, Coptoborus* and *Theoborus*) and the clade of widespread *Xyleborus* species may indicate that the Neotropical region is important in their evolutionary history [72]. However, some of the closest relatives of our focal species are endemic to the Afrotropical region (e.g. *X. alluaudi* and related species) [72], which complicates the inference of biogeographical history from such higher level taxonomy, and highlights that dispersal, not vicariance, is likely the primary driver of biogeographical structure in this group. Our biogeographical analyses support the Neotropical association in three of the focal species (ambiguous in *X. perforans*). It should be pointed out, however, that the reconstructed region for *X. affinis* seems to be strongly affected by the underlying node. Since the topology at this node has poor support (posterior probability=0.87), it may be unwise to emphasize this state when determining the ancestral region. This, together with the high haplotypic diversity in the Afrotropics, suggests an Afrotropical origin isfor *X. affinis*.

**Fig. 3:**
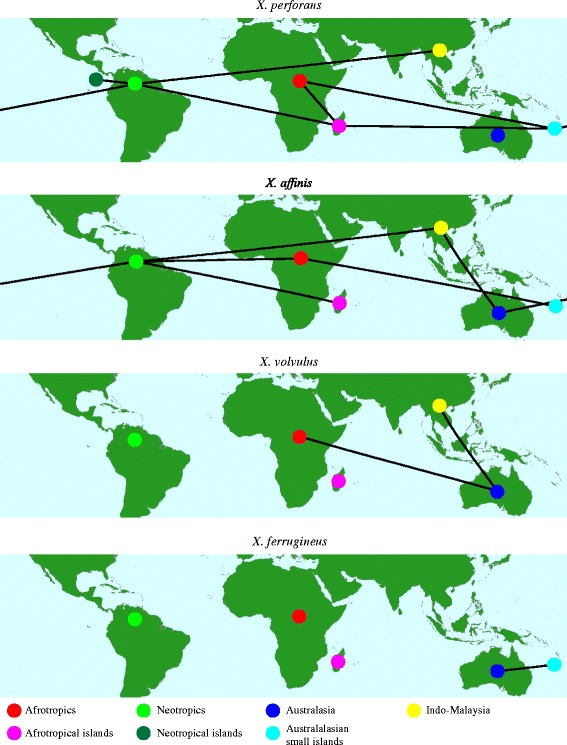
Supported migration routes. Legend: Maps show strongly supported diffusions (Bayes factor <10) from separate biogeographic reconstructions for the four monophyletic focal species constructed using COI and EF1α

Differences in dispersal rates among the focal species were illustrated by the high number of unconnected locations for *X. ferrugineus* in the SPREAD analysis, as compared to *X. affinis* and *X. perforans* in particular (Fig. 3). This is unsurprising given the low occurrence of recent introductions to new regions for *X. ferrugineus*, as evident from the limited number of shared or similar haplotypes between regions (Fig. 1; haplotype network). High haplotypic diversity within multiple regions (Fig. 1) furthermore suggests enduring populations of this species, only seen elsewhere in the Afrotropical clade of *X. affinis*. This interpretation is supported by the distribution of node ages among the four species in the phylogeny: the nodes separating lineages in *X. ferrugineus* are substantially older than those observed in the other three species, as also reflected in the genetic distances between alleles in the COI mismatch distribution plots (Additional file 1: Figure S3). It should be mentioned that the EF1α mismatch distributions were similar among all four species, which is likely due to EF1α not having captured the most recent divergences. This difference in resolution between COI and EF1α can be seen in their haplotype networks (Fig. 1, and Additional file 1: Figure S4).Why does *X. ferrugineus* exhibit these largely unique characteristics? In particular, why have its ancient and geographically pure clades not been eroded by the recent spread and establishment seen in the other three species? Incomplete or biased sampling of individuals may limit the potential detection of more recently introduced lineages. However, that such lineages should be excluded by chance alone seems unlikely given the high representation of new introductions in the other species and the fact that *X. ferrugineus* has the second highest sample size (53 individuals). Some of these results (higher genetic diversity, deeper phylogenetic divergences) can be explained by *X. ferrugineus* simply being older than its three related species. Why this species seems largely unaffected by anthropogenic effects—in stark contrast to the other *Xyleborus* species—is a question that deserves further study at a finer scale.

Given that ancient long-range dispersal has occurred repeatedly in this clade of *Xyleborus*, it may not be unrealistic to expect cryptic speciation in the absence of sexual selection [70, 73]. Several previous synonyms of *X. ferrugineus* has recently been resurrected to full species status [71, 74], which makes us alert to this possibility. We note that the New Caledonia population, which likely constitutes a very old dispersal event from the Neotropics, was separate from the other populations in both data sets (COI and EF1α; Additional file 1: Figure S2), but without any morphological differentiation. *Xyleborus bispinatus* and *X. impressus,* which were previously considered as *X. ferrugineus,* can on the other hand be readily distinguished genetically and morphologically and hence they are more clearly separated than the New Caledonia population. It is furthermore possible that broader geographical sampling of *X. ferrugineus* also will reduce some of the genetic gaps observed between populations of this species. Questions have been raised about the validity of species status of the near identical *X. volvulus* and *X. perforans* [45, 75]. Our data nevertheless solved these issues confidently and we conclude that *X. volvulus* and *X. perforans* represent two individual species—both with regard to phylogenetic monophyly, and to their separate biogeographical origins. However, they are both globally widespread, and not restricted to the Neotropics and the Old World, respectively, as previously assumed [44].

## Conclusion

We have tested the hypothesis that the global distributions in many *Xyleborus* species were fostered by human transportation. We found ample evidence for recent colonisations of new regions in three of the four examined *Xyleborus* species, which is in line with this hypothesis. However, the genetic data also strongly suggest that all four species were present across most of their current distributional range several million years ago, which was clearly before humans crossing the oceans. The combination of a pre-adapted biology for propagule establishment far from the source population, and the frequent transportation of wood material by humans in modern times, has radically increased migration rates in these beetles. It is therefore not surprising that many species of *Xyleborus* are among the most frequently collected species in any tropical or subtropical region.

## Availability of supporting data

Supporting figures and tables are found in the additional file. All analysed DNA sequences are available in Genbank. COI acc.no: KP941137-KP941327), EF1α acc.no: KP941328-KP941418, accession numbers for ingroup and outgroup sequences are listed in (Additional file 1: Table S3).

## Additional file

Additional file 1: Supplemental tables and figures: **Table S1.**—Collected data and sequencing coverage for COI and EF1α. **Table S2.**—Sequencing primers. **Table S3.**—Out and ingroup specimen collection data, with Genbank accession numbers. **Table S4.**—Evolutionary models and rates, and summary statistics including ESS values from the biogeographic and phylogenetic reconstruction shown in Fig. 1. **Table S5.**—Evolutionary models and rates, and summary statistics including ESS values from the species level biogeographic and phylogenetic reconstructions used for the SPREAD plots. **Figure S1.**—All specimens, for which we had coordinates, plotted on a word map. **Figure S2.**—A tanglegram showing the level of concordance between the two phylogenetic markers (COI and EF1α). **Figure S3.**—Mismatch distribution plots showing the distribution of distances between alleles expected under stable population size and the empirical data. **Figure S4.**—EF1α haplotype network. **Figure S5.**—Mantel tests of genetic and geographic distance. (PDF 2578 kb)
