# Supplementary material for: Globally distributed Xyleborus species reveal recurrent intercontinental dispersal in a landscape of ancient worldwide distributions
Source: BMC Evol Biol. 2016 Feb 15;16:37. doi: 10.1186/s12862-016-0610-7 (PMC4753646; doi:10.1186/s12862-016-0610-7)
Supplement: Additional file 1: — Supplemental tables and figures: Table S1.—Collected data and sequencing coverage for COI and EF1α. Table S2.—Sequencing primers. Table S3.—Out and ingroup specimen collection data, with Genbank accession numbers. Table S4.—Evolutionary models and rates, and summary statistics including ESS values from the biogeographic and phylogenetic reconstruction shown in Fig. 1. Table S5.—Evolutionary models and rates, and summary statistics including ESS values from the species level biogeographic and phylogenetic reconstructions used for the SPREAD plots. Figure S1.—All specimens, for which we had coordinates, plotted on a word map. Figure S2.—A tanglegram showing the level of concordance between the two phylogenetic markers (COI and EF1α). Figure S3.—Mismatch distribution plots showing the distribution of distances between alleles expected under stable population size and the empirical data. Figure S4.—EF1α haplotype network. Figure S5.—Mantel tests of genetic and geographic distance. (PDF 2578 kb) [file 12862_2016_610_MOESM1_ESM.pdf]

BMC Evolutionary Biology

SUPPORTING INFORMATION

Globally distributed *Xyleborus* species reveal recurrent intercontinental dispersal in a landscape of ancient worldwide distributions

Authors: Jostein Gohli, Tina Selvarajah, Lawrence R. Kirkendall, Bjarte H. Jordal

Appendix S1

Table S1 – Collected data and sequence coverage for COI and EF1α. Region codes are as follows: AF = Africa, AF(IO) = Africa (Indian Ocean), AM = America, AM(PO) = America (Pacific Ocean), IM = Indo-Malaysia, OC = Oceania, OC(PO) = Oceania (Pacific Ocean).

	Species	Alias	Locality	Country	Region	Longitude, Latitude	COI sequenced	EF1α sequenced
1	*Xyleborus affinis*	aff02CR	La Selva Biological Station	Costa Rica	AM	10.424, -84.013	yes	yes
2	*Xyleborus affinis*	aff03CR	La Selva Biological Station	Costa Rica	AM	10.424, -84.013	yes	yes
3	*Xyleborus affinis*	aff04CR	Golfito	Costa Rica	AM	8.637, -83.158	yes	yes
4	*Xyleborus affinis*	aff05CR	Golfito	Costa Rica	AM	8.637, -83.158	yes	yes
5	*Xyleborus affinis*	aff06UG	Kibale Forest N.P.	Uganda	AF	0.484, 30.412	yes	
6	*Xyleborus affinis*	aff07UG	Kibale Forest N.P.	Uganda	AF	0.484, 30.412	yes	yes
7	*Xyleborus affinis*	aff08UG	Kibale Forest N.P.	Uganda	AF	0.484, 30.412	yes	
8	*Xyleborus affinis*	aff09MY	Lambir Hills N.P., Sarawak	Malaysia	IM	4.201, 114.039	yes	
9	*Xyleborus affinis*	aff10MY	Lambir Hills N.P., Sarawak	Malaysia	IM	4.201, 114.039	yes	
10	*Xyleborus affinis*	aff12TH	Rai Lai	Thailand	IM	8.014, 98.840	yes	
11	*Xyleborus affinis*	aff16PG	Ohu, Madang	Papua New Guinea	OC	-5.229,145.702	yes	
12	*Xyleborus affinis*	aff17MY	Danum Valley, Sabah	Malaysia	IM	4.822, 117.676	yes	
13	*Xyleborus affinis*	aff18PG	Utai, East Sepik Province	Papua New Guinea	OC	-3.31, 141.59	yes	
14	*Xyleborus affinis*	aff19PG	Oro, Popondetta Chiki	Papua New Guinea	OC	-8.810, 148.217	yes	
15	*Xyleborus affinis*	aff20MX	Chiapas, Tapachula	Mexico	AM	14.963, -92.244	yes	yes

16	*Xyleborus affinis*	aff21MX	Córdoba, Veracruz	Mexico	AM	18.901, -96.920	yes	
17	*Xyleborus affinis*	aff22MX	El Palomar, Edo	Mexico	AM		yes	yes
18	*Xyleborus affinis*	aff23MX	Jungapeo, Michoacan	Mexico	AM	19.494, -100.491	yes	
19	*Xyleborus affinis*	aff24MX	Guerrero, Santa Fe	Mexico	AM	17.246, -100.533	yes	
20	*Xyleborus affinis*	aff25MX	Guerrero, Santa Fe	Mexico	AM	17.246, -100.533	yes	yes
21	*Xyleborus affinis*	aff26GU	Iwokrama F.S.	Guyana	AM	5.004, -58.919	yes	yes
22	*Xyleborus affinis*	aff27GU	Iwokrama F.S.	Guyana	AM	5.004, -58.919	yes	
23	*Xyleborus affinis*	aff29GU	Iwokrama F.S.	Guyana	AM	5.004, -58.919	yes	yes
24	*Xyleborus affinis*	aff30NC	Poquereux	New Caledonia	OC (PO)	-21.697, 165.894	yes	yes
25	*Xyleborus affinis*	aff32NC	Poquereux	New Caledonia	OC (PO)	-21.697, 165.894	yes	
26	*Xyleborus affinis*	aff34GH	Bia N.P.	Ghana	AF	6.496, -3.075	yes	yes
27	*Xyleborus affinis*	aff35GH	Cape 3 points	Ghana	AF	4.756, -2.099	yes	yes
28	*Xyleborus affinis*	aff36GH	Ankasa Game Reserve & Nini-Suhien N.P.	Ghana	AF	5.273, -2.556	yes	yes
29	*Xyleborus affinis*	aff37GH	Ankasa Game Reserve & Nini-Suhien N.P.	Ghana	AF	5.273, -2.556	yes	yes
30	*Xyleborus affinis*	aff38GH	Bokuro-Abaa	Ghana	AF		yes	yes
31	*Xyleborus affinis*	aff39GH	Ankasa Game Reserve & Nini-Suhien N.P.	Ghana	AF	5.273, -2.556	yes	yes
32	*Xyleborus affinis*	aff40GH	Ankasa Game Reserve & Nini-Suhien N.P.	Ghana	AF	5.273, -2.556	yes	yes
33	*Xyleborus affinis*	aff41GH	Ankasa Game Reserve & Nini-Suhien N.P.	Ghana	AF	5.273, -2.556	yes	yes
34	*Xyleborus affinis*	aff43MA	Fôret Classée d'Analavelona	Madagascar	AF(IO)	-23.120, 44.534	yes	
35	*Xyleborus affinis*	aff44MA	Réserve Spéciale de l'Ankarana	Madagascar	AF(IO)	-12.912, 49.118	yes	
36	*Xyleborus affinis*	aff45MA	Nosy Be, Réserve Naturelle Intégrale de Lokobe	Madagascar	AF(IO)	-13.389, 48.283	yes	
37	*Xyleborus affinis*	aff46MA	Parc National d'Ankarafantsika, Ampijoroa Station Forestière	Madagascar	AF(IO)	-16.162, 47.115	yes	
38	*Xyleborus affinis*	aff48MA	Montagne d'Anjanaharibe	Madagascar	AF(IO)	-21.300, 44.885	yes	
39	*Xyleborus affinis*	aff50TH	Unknown	Thailand	IM		yes	yes
40	*Xyleborus affinis*	aff51TH	Unknown	Thailand	IM		yes	yes
41	*Xyleborus affinis*	aff52MX	Chiapas, Tapachula	Mexico	AM	14.963, -92.244	yes	yes
42	*Xyleborus affinis*	aff53MX	Guerero, Santa Fe	Mexico	AM	17.246, -100.533	yes	yes
43	*Xyleborus affinis*	aff54PG	Ohu, Madang	Papua New Guinea	OC	-5.229,145.702	yes	yes
44	*Xyleborus affinis*	aff55MY	Danum Valley, Sabah	Malaysia	IM	4.822, 117.676	yes	yes
45	*Xyleborus affinis*	aff56TH	Nam Nao N.P.	Thailand	IM	16.759, 101.586	yes	yes
46	*Xyleborus affinis*	aff57MY	Danum Valley, Sabah	Malaysia	IM	4.822, 117.676	yes	

47	*Xyleborus affinis*	aff59GU	Iwokrama F.S.	Guyana	AM	5.004, -58.919	yes	
48	*Xyleborus affinis*	aff60SC	Mahe	Seychelles	AF(IO)	-4.632, 55.438	yes	yes
49	*Xyleborus affinis*	aff61SC	Mahe	Seychelles	AF(IO)	-4.632, 55.438	yes	yes
50	*Xyleborus affinis*	aff62CA	Limbe Botanical Garden	Cameroon	AF	4.016, 9.203	yes	
51	*Xyleborus affinis*	aff63CA	Ekanjo, Limbe	Cameroon	AF	4.072, 9.175	yes	
52	*Xyleborus affinis*	aff64CA	Ekanjo, Limbe	Cameroon	AF	4.072, 9.175	yes	yes
53	*Xyleborus affinis*	aff67CA	Bonadi kombe, Limbe	Cameroon	AF	3.998, 9.263	yes	yes
54	*Xyleborus affinis*	aff70PE	Huanuco	Peru	AM	-9.398, -76.212	yes	
55	*Xyleborus affinis*	aff73PA	San Lorenzo Forest, Colón Province.	Panama	AM	9.17,- 79.58	yes	
56	*Xyleborus affinis*	aff74PA	San Lorenzo Forest, Colón Province	Panama	AM	9.17,- 79.58	yes	
57	*Xyleborus affinis*	aff75CA	Limbe Botanical Garden	Cameroon	AF	4.016, 9.203	yes	
58	*Xyleborus affinis*	aff76CA	Limbe Botanical Garden	Cameroon	AF	4.016, 9.203	yes	
59	*Xyleborus affinis*	aff77CA	Limbe Botanical Garden	Cameroon	AF	4.016, 9.203	yes	
60	*Xyleborus affinis*	aff78CA	Ekanjo, Limbe	Cameroon	AF	4.072, 9.175	yes	
61	*Xyleborus affinis*	aff82BR	Barra do Cachoeira, Parana	Brazil	AM	-25.586, -51.443	yes	
62	*Xyleborus affinis*	aff83BR	Barra do Cachoeira, Parana	Brazil	AM	- 25.586, -51.443	yes	
63	*Xyleborus ferrugineus*	fer05MX	Guerrero, Santa Fe	Mexico	AM	17.246, -100.533	yes	
64	*Xyleborus ferrugineus*	fer06NC	Poquereux	New Caledonia	OC (PO)	-21.697, 165.894	yes	yes
65	*Xyleborus ferrugineus*	fer09GU	Iwokrama F.S.	Guyana	AM	5.004, -58.919	yes	yes
66	*Xyleborus ferrugineus*	fer11GH	Kakum N.P.	Ghana	AF	5.428, -1.320	yes	
67	*Xyleborus ferrugineus*	fer12GH	Bia N.P.	Ghana	AF	6.496, -3.075	yes	yes
68	*Xyleborus ferrugineus*	fer13GH	Cape 3 points	Ghana	AF	4.756, -2.099	yes	yes
69	*Xyleborus ferrugineus*	fer14GH	Cape 3 points	Ghana	AF	4.756, -2.099	yes	yes
70	*Xyleborus ferrugineus*	fer15GH	Ankasa Game Reserve & Nini-Suhien N.P.	Ghana	AF	5.273, -2.556	yes	
71	*Xyleborus ferrugineus*	fer16GH	Bokuro-Abaa	Ghana	AF		yes	yes
72	*Xyleborus ferrugineus*	fer17GH	Ankasa Game Reserve & Nini-Suhien N.P.	Ghana	AF	5.273, -2.556	yes	yes
73	*Xyleborus ferrugineus*	fer18GH	Ankasa Game Reserve & Nini-Suhien N.P.	Ghana	AF	5.273, -2.556	yes	
74	*Xyleborus ferrugineus*	fer19GH	Ankasa Game Reserve & Nini-Suhien N.P.	Ghana	AF	5.273, -2.556	yes	
75	*Xyleborus ferrugineus*	fer21MA	Forêt de Tsimembo	Madagascar	AF(IO)	-19.143, 44.809	yes	
76	*Xyleborus ferrugineus*	fer22MA	Parc National de Kirindy Mitea	Madagascar	AF(IO)	-20.694, 44.198	yes	
77	*Xyleborus ferrugineus*	fer23MA	Parc National Tsingy de Bemaraha	Madagascar	AF(IO)	-20.694, 44.198	yes	

78	*Xyleborus ferrugineus*	fer24MA	Montagne d'Anjanaharibe	Madagascar	AF(IO)	-21.300, 44.885	yes	
79	*Xyleborus ferrugineus*	fer25MA	Unknown	Madagascar	AF(IO)		yes	yes
80	*Xyleborus ferrugineus*	fer26MX	Jungapeo, Michoacán	Mexico	AM	19.494, -100.491	yes	
81	*Xyleborus ferrugineus*	fer27PG	Ohu, Madang	Papua New Guinea	OC	-5.229,145.702	yes	
82	*Xyleborus ferrugineus*	fer28MA	Forêt de Tsimembo	Madagascar	AF(IO)	-19.143, 44.809	yes	
83	*Xyleborus ferrugineus*	fer29MA	Parc National Tsingy de Bemaraha	Madagascar	AF(IO)	18.672, 44.748	yes	
84	*Xyleborus ferrugineus*	fer31PG	Ohu, Madang	Papua New Guinea	OC	-5.229,145.702	yes	yes
85	*Xyleborus ferrugineus*	fer36GH	Unknown	Ghana	AF		yes	yes
86	*Xyleborus ferrugineus*	fer37GH	Unknown	Ghana	AF		yes	
87	*Xyleborus ferrugineus*	fer38PG	Ohu, Madang	Papua New Guinea	OC	-5.229,145.702	yes	yes
88	*Xyleborus ferrugineus*	fer39PG	Ohu, Madang	Papua New Guinea	OC	-5.229,145.702	yes	
89	*Xyleborus ferrugineus*	fer41GU	Unknown	Guyana	AM		yes	
90	*Xyleborus ferrugineus*	fer44CR	Unknown	Costa Rica	AM		yes	
91	*Xyleborus ferrugineus*	fer45SC	Mahe	Seychelles	AF(IO)	-4.632, 55.438	yes	yes
92	*Xyleborus ferrugineus*	fer46CR	La Virgen	Costa Rica	AM	10.406, -84.137	yes	yes
93	*Xyleborus ferrugineus*	fer52CA	Ekanjo, Limbe	Cameroon	AF	4.072, 9.175	yes	
94	*Xyleborus ferrugineus*	fer53CA	Ekanjo, Limbe	Cameroon	AF	4.072, 9.175	yes	
95	*Xyleborus ferrugineus*	fer54CA	Ekanjo, Limbe	Cameroon	AF	4.072, 9.175	yes	yes
96	*Xyleborus ferrugineus*	fer55CA	Bimbia	Cameroon	AF	3.954, 9.244	yes	
97	*Xyleborus ferrugineus*	fer56CA	Bonadi kombe, Limbe	Cameroon	AF	3.998, 9.263	yes	
98	*Xyleborus ferrugineus*	fer57CA	Bakingli, Limbe	Cameroon	AF	4.083, 9.053	yes	yes
99	*Xyleborus ferrugineus*	fer58PA	San Lorenzo Forest, Colón Province	Panama	AM	9.17,- 79.58	yes	yes
100	*Xyleborus ferrugineus*	fer60PA	San Lorenzo Forest, Colón Province	Panama	AM	9.17,- 79.58	yes	
101	*Xyleborus ferrugineus*	fer61SC	Mahe	Seychelles	AF(IO)	-4.632, 55.438	yes	yes
102	*Xyleborus ferrugineus*	fer62SC	Mahe	Seychelles	AF(IO)	-4.632, 55.438	yes	
103	*Xyleborus ferrugineus*	fer63CA	Limbe Botanical Garden	Cameroon	AF	4.016, 9.203	yes	yes
104	*Xyleborus ferrugineus*	fer64CA	Bakingli, Limbe	Cameroon	AF	4.083, 9.053	yes	
105	*Xyleborus ferrugineus*	fer65CA	Bakingli, Limbe	Cameroon	AF	4.083, 9.053	yes	yes
106	*Xyleborus ferrugineus*	fer66CA	Bonadi Kombe, Limbe	Cameroon	AF	3.998, 9.263	yes	
107	*Xyleborus ferrugineus*	fer70BR	Barra do Cachoeira, Parana	Brazil	AM	- 25.586, -51.443	yes	
108	*Xyleborus ferrugineus*	fer72HO	Unknown	Honduras	AM		yes	

109	*Xyleborus ferrugineus*	fer73CR	Unknown	Costa Rica	AM		yes	
110	*Xyleborus ferrugineus*	fer74NC	Poquereux	New Caledonia	OC (PO)	-21.697, 165.894	yes	
111	*Xyleborus ferrugineus*	fer75NC	Poquereux	New Caledonia	OC (PO)	-21.697, 165.894	yes	
112	*Xyleborus ferrugineus*	fer76CR	Unknown	Costa Rica	AM		yes	
113	*Xyleborus ferrugineus*	fer998CR	Unknown	Costa Rica	AM		yes	
114	*Xyleborus ferrugineus*	fer999CR	Unknown	Costa Rica	AM		yes	
115	*Xyleborus ferrugineus*	ferHO	Unknown	Honduras	AM		yes	
116	*Xyleborus perforans*	per01PG	Confiscated at a port	Papua New Guinea	OC		yes	
117	*Xyleborus perforans*	per02JP	Japan, Ishigaki	Japan	IM	24.406, 124.186	yes	
118	*Xyleborus perforans*	per03PG	Lae, Morobe Province	Papua New Guinea	OC	-6.723, 146.991	yes	
119	*Xyleborus perforans*	per04PG	Mes	Papua New Guinea	OC	-9.407, 147.166	yes	
120	*Xyleborus perforans*	per05PG	Morobe, Wau	Papua New Guinea	OC	-7.340, 146.706	yes	
121	*Xyleborus perforans*	per06PG	Morobe, Wau	Papua New Guinea	OC	-7.340, 146.706	yes	yes
122	*Xyleborus perforans*	per07MY	Lambir Hills NP, Sarawak	Malaysia	IM	4.201, 114.039	yes	
123	*Xyleborus perforans*	per08MY	Lambir Hills NP, Sarawak	Malaysia	IM	4.201, 114.039	yes	
124	*Xyleborus perforans*	per13TH	Rai Lai	Thailand	IM	8.014, 98.840	yes	
125	*Xyleborus perforans*	per17IC	Unknown	Cocos island	AM(PO)	5.528, -87.067	yes	yes
126	*Xyleborus perforans*	per18IC	Unknown	Cocos island	AM(PO)	5.528, -87.067	yes	
127	*Xyleborus perforans*	per25PG	Ohu, Madang	Papua New Guinea	OC	-5.229,145.702	yes	
128	*Xyleborus perforans*	per35PG	Oro, Popondetta Chiki	Papua New Guinea	OC	-8.810, 148.217	yes	
129	*Xyleborus perforans*	per37NC	Poquereux	New Caledonia	OC (PO)	-21.697, 165.894	yes	
130	*Xyleborus perforans*	per38NC	Poquereux	New Caledonia	OC (PO)	-21.697, 165.894	yes	yes
131	*Xyleborus perforans*	per39GH	Bokuro-Abaa	Ghana	AF		yes	
132	*Xyleborus perforans*	per39NC	Poquereux	New Caledonia	OC (PO)	-21.697, 165.894	yes	
133	*Xyleborus perforans*	per41MA	Parc National d'Ankarafantsika	Madagascar	AF(IO)	-16.162, 47.115	yes	
134	*Xyleborus perforans*	per49PG	Oro, Popondetta Chiki	Papua New Guinea	OC	-8.810, 148.217	yes	yes
135	*Xyleborus perforans*	per52BG	Khulna Paper Mill	Bangladesh	IM	22.804, 89.552	yes	yes
136	*Xyleborus perforans*	per53TH	Unknown	Thailand	IM		yes	yes
137	*Xyleborus perforans*	per59MA	Parc National d'Ankarafantsika	Madagascar	AF(IO)	-16.162, 47.115	yes	
138	*Xyleborus perforans*	per61JP	Japan, Ishigaki	Japan	IM	24.406, 124.186	yes	
139	*Xyleborus perforans*	per62CR	La Gamba Field Station	Costa Rica	AM	8.700, -83.201	yes	

140	*Xyleborus perforans*	per62SC	Mahe	Seychelles	AF(IO)	-4.632, 55.438	yes	
141	*Xyleborus perforans*	per63SC	Unknown	Seychelles	AF(IO)		yes	yes
142	*Xyleborus perforans*	per64SC	Unknown	Seychelles	AF(IO)		yes	yes
143	*Xyleborus perforans*	per66SC	Unknown	Seychelles	AF(IO)		yes	
144	*Xyleborus perforans*	per67AU	Queensland, Babinda	Australia	OC	-17.341, 145.893	yes	
145	*Xyleborus perforans*	per74CA	Limbe, Botanical Garden	Cameroon	AF	4.016, 9.203	yes	yes
146	*Xyleborus volvolus*	vol12CR	Cahuita, Limón	Costa Rica	AM	9.731, -82.827	yes	yes
147	*Xyleborus volvolus*	vol14GH	Unknown	Ghana	AF		yes	yes
148	*Xyleborus volvolus*	vol16GH	Unknown	Ghana	AF		yes	
149	*Xyleborus volvolus*	vol32MX	Jungapeo, Michoacan	Mexico	AM	19.494, -100.491	yes	
150	*Xyleborus volvolus*	vol35MX	Guerrero, Santa Fe	Mexico	AM	17.246, -100.533	yes	
151	*Xyleborus volvolus*	vol36MX	Guerrero, Santa Fe	Mexico	AM	17.246, -100.533	yes	yes
152	*Xyleborus volvolus*	vol37GH	Bia N.P.	Ghana	AF	6.496, -3.075	yes	
153	*Xyleborus volvolus*	vol38GH	Bokuro-Abaa	Ghana	AF		yes	yes
154	*Xyleborus volvolus*	vol40GH	Ankasa Game Reserve & Nini-Suhien N.P.	Ghana	AF	5.273, -2.556	yes	yes
155	*Xyleborus volvolus*	vol43MA	Parc National d'Ankarafantsika	Madagascar	AF(IO)	-16.162, 47.115	yes	
156	*Xyleborus volvolus*	vol45MX	Tapachula, Chiapas	Mexico	AM	14.963, -92.244	yes	yes
157	*Xyleborus volvolus*	vol46MX	Tapachula, Chiapas	Mexico	AM	14.963, -92.244	yes	yes
158	*Xyleborus volvolus*	vol47MX	Guerero, Santa Fe	Mexico	AM	17.246, -100.533	yes	
159	*Xyleborus volvolus*	vol48MX	Guerero, Santa Fe	Mexico	AM	17.246, -100.533	yes	yes
160	*Xyleborus volvolus*	vol53PG	Ohu, Madang	Papua New Guinea	OC	-5.229,145.702	yes	
161	*Xyleborus volvolus*	vol54PG	Ohu, Madang	Papua New Guinea	OC	-5.229,145.702	yes	yes
162	*Xyleborus volvolus*	vol55MX	Tapachula, Chiapas	Mexico	AM	14.963, -92.244	yes	
163	*Xyleborus volvolus*	vol56MX	Tapachula, Chiapas	Mexico	AM	14.963, -92.244	yes	yes
164	*Xyleborus volvolus*	vol57MX	Tapachula, Chiapas	Mexico	AM	14.963, -92.244	yes	
165	*Xyleborus volvolus*	vol58CR	La Gamba Field Station	Costa Rica	AM	8.700, -83.201	yes	yes
166	*Xyleborus volvolus*	vol60BG	Supati Forest Station, Sundarbaans	Bangladesh	IM	22.048, 89.817	yes	
167	*Xyleborus volvolus*	vol60TH	Rai Lai	Thailand	IM	8.014, 98.840	yes	yes
168	*Xyleborus volvolus*	vol61CR	CATIE, Turrialba	Costa Rica	AM	9.902, -83.684	yes	yes
169	*Xyleborus volvolus*	vol71CA	Bakingli, Limbe	Cameroon	AF	4.083, 9.053	yes	
170	*Xyleborus volvolus*	vol73PA	San Lorenzo Forest, Colón Province	Panama	AM	9.17,- 79.58	yes	

Table S2 – Sequencing primers (Normark *et al.* 1999).

Gene	Primer	F/R	Sequence (5’–3’)
COI	s1718	f	5'-GGA GGA TTT GGA AAT TGA TTA GTT CC-3'
	a2411	r	5'-GCT AAT CAT CTA AAA ACT TTA ATT CCW GTW G-3'
	a2237	r	5'-CCG AAT GCT TCT TTT TTA CCT CTT TCT TG-3'
EF-1α	efs149	F	f 5’-ATC GAG AAG TTC GAG AAG GAG GCY CAR GAA ATG GG-3’
	efa1043	R	r 5’-GTA TAT CCA TTG GAA ATT TGA CCN GGR TGR TT -3’
	efa754	r	5’-CCA CCA ATT TTG TAG ACA TC-3’

Table S3 – Out- and ingroup specimen collection data, with Genbank accession numbers. The bolded specimens were used in only the dating analysis.

SPECIES	Locality	Country	Region	Longitude, Latitude	Acc. COI	Acc. EF-1α
Xyleborus multispinatus	Kibale, Fort Portal	Uganda	AF		AF187140	AF186690
Coccotrypes cyperi	Unknown	Costa Rica	AM		AF375309	AF259863
Ozopemon brownie	Bako NP, Sarawak	Borneo	IM		AF438514	AF259870
Coccotrypes advena	Kibale, Fort Portal	Uganda	AF		AF444056	AF444072
Coccotrypes cyperi	Cairns, Queensland	Australia	OC		AF444058	AF444074
Coccotrypes advena	La Selva, Puerto de la Viejo	Costa Rica	AM		AF444062	AF444076
Xylosandrus morigerus	Unknown	Thailand	IM		JN982494	GU808754
Xyleborus bispinatus	La Virgen	Costa Rica	AM	10.406, -84.137	KP941316	-
Xyleborus bispinatus	Iwokrama F.S.	Guyana	AM	5.004, -58.919	KP941311	-
Xyleborus bispinatus	Unknown	Costa Rica	AM		KP941317	-
Xyleborus bispinatus	Unknown	Brazil	AM		KP941310	-
Xyleborus bispinatus	Unknown	Costa Rica	AM		KP941313	-
Xyleborus bispinatus	Iwokrama F.S.	Guyana	AM	5.004, -58.919	KP941312	-
Xyleborus bispinatus	San Lorenzo Forest, Colón Province	Panama	AM	9.17,- 79.58	KP941314	-
Xyleborus semipunctatus	La Selva, Puerto de la Viejo	Costa Rica	AM		KP941323	-
Xyleborus sparsipilosus	La Virgen (pinned specimen)	Costa Rica	AM	10.406, -84.137	KP941322	KP941417
Xyleborus bolivianus		Costa Rica	AM		KP941322	-
Xyleborus cognatus	Madang	Papua New Guinea	OC		KP941319	-
Xyleborus cognatus	Madang	Papua New Guinea	OC		KP941320	KP941415
Xyleborus cognatus	Sunderbaans	Bangladesh	IM		KP941321	KP941416
Xyleborus sp. 1	La Selva, Puerto de la Viejo	Thailand	IM		KP941324	KP941418
Xyleborus sp. 2	La Selva, Puerto de la Viejo	Costa Rica	AM		KP941325	-
Xyleborus sp. 3	Iwokrama F.S.	Guyana	AM		KP941326	-
Xyleborus sp. 4	Iwokrama F.S.	Guyana	AM		KP941327	-
Xyleborus alluadi					HM064118.1	
Sanosonius dampfi					HM064095.1	AF259885.1
Theoborus sp. A					HM064101.1	
Theoborus sp.B					HM064100.1	HM064194.1
Coptoborus pseudotenius					HM064071.1	AF508880.1
Coptoborus sp. 558					HM064072.1	HM064172.1

Table S4 – Evolutionary models and rates, and summary statistics including ESS values from the biogeographic and phylogenetic reconstruction shown in figure 1.

COI - evolutionary model and rates	HKY+G+I	
EF1α - evolutionary model and rates	TN93+G	
**Statistic**	**Mean**	**ESS**
Prior	319.4	373.8
Likelihood	-10586.3	1610.7
treeModel.rootHeight	0.2	621.8
yule.birthRate	94.0	517.5
COI.kappa	10.2	1820.8
COI.frequencies1	0.3	1336.7
COI.frequencies2	0.1	1242.6
COI.frequencies3	0.2	1297.5
COI.frequencies4	0.4	1074.5
COI.alpha	1.0	2247.6
COI.pInv	0.6	2188.2
EF1a.kappa1	5.6	2085.5
EF1a.kappa2	12.9	1917.9
EF1a.frequencies1	0.3	1188.7
EF1a.frequencies2	0.2	1608.1
EF1a.frequencies3	0.2	1316.8
EF1a.frequencies4	0.3	1277.4
EF1a.alpha	0.1	1282.6
COI.clock.rate	2.6	557.9
EF1a.clock.rate	0.2	562.4
GEO.clock.rate	11.9	632.1
GEO.rates1	0.9	5879.8
GEO.rates2	1.9	6412.4
GEO.rates3	1.0	5917.7
GEO.rates4	0.9	5875.5
GEO.rates5	1.0	5573.5
GEO.rates6	1.0	5549.8
GEO.rates7	1.2	6756.3
GEO.rates8	1.0	6040.1
GEO.rates9	1.0	5976.1
GEO.rates10	1.0	5103.7
GEO.rates11	0.9	5256.7
GEO.rates12	1.0	5558.3

GEO.rates13	1.1	2384.8
GEO.rates14	1.2	6124.5
GEO.rates15	0.7	5958.0
GEO.rates16	1.0	5609.3
GEO.rates17	0.5	6292.8
GEO.rates18	0.9	5827.4
GEO.rates19	1.1	5647.5
GEO.rates20	1.0	5716.6
GEO.rates21	0.7	6551.6
GEO.indicators1	0.0	1593.5
GEO.indicators2	1.0	-
GEO.indicators3	0.0	6084.0
GEO.indicators4	0.2	6480.1
GEO.indicators5	0.1	5831.3
GEO.indicators6	0.0	5937.2
GEO.indicators7	1.0	-
GEO.indicators8	0.0	6825.0
GEO.indicators9	1.0	6825.0
GEO.indicators10	0.0	6825.0
GEO.indicators11	0.1	1774.0
GEO.indicators12	0.0	6164.5
GEO.indicators13	1.0	6013.5
GEO.indicators14	1.0	6825.0
GEO.indicators15	0.7	3589.6
GEO.indicators16	0.0	6464.7
GEO.indicators17	1.0	6825.0
GEO.indicators18	0.0	6825.0
GEO.indicators19	1.0	3091.1
GEO.indicators20	0.1	6719.9
GEO.indicators21	0.9	6456.5
GEO.nonZeroRates	9.3	5597.4
COI.treeLikelihood	-7843.7	636.2
EF1a.treeLikelihood	-2578.0	1941.3
GEO.treeLikelihood	-164.5	343.9
Speciation	614.4	376.2

Table S5 – Evolutionary models and rates, and summary statistics including ESS values from the species level biogeographic and phylogenetic reconstructions used for the SPREAD plots.

	X. perforans		X. volvulus		X. ferrugineus		X. affinis	
COI - evolutionary model and rates	HKY+G+I		HKY+G		HKY+G-I		TN93+G+I	
EF1α - evolutionary model and rates	TN93		HKY		HKY		HKY	
Tracer statistic	Mean	ESS	Mean	ESS	Mean	ESS	Mean	ESS
posterior	-2082.135	1641	-1666.963	1362	-2540.529	1412	-1747.825	1141
prior	-138.795	1928	-98.444	1337	-128.707	1392	-171.17	1125
likelihood	-1943.34	2896	-1568.519	2998	-2411.822	2216	-1576.655	1758
treeModel.rootHeight	26.815	1902	37.404	1794	5.631	1121	9.716	1080
constant.popSize	14.851	1893	11.113	1868	5.496	1180	6.024	1054
kappa	7.09	2235	6.522	2148	10.987	2463	17.964	1784
frequencies1	0.314	1248	0.332	1134	0.325	1139	0.296	1192
frequencies2	0.154	1177	0.14	1130	0.135	870	0.166	1283
frequencies3	0.242	1124	0.241	1088	0.273	1004	0.228	905
frequencies4	0.29	916	0.287	1217	0.268	1060	0.31	1147
clock.rate	7.27E-03	2231	6.40E-03	1729	3.37E-02	1444	1.01E-02	1341
region.clock.rate	0.666	2689	0.667	2799	0.657	2411	0.671	2141
region.rates1	0.9	598	0.778	902	0.714	937	0.576	823
region.rates2	1.116	745	1.332	1631	3.509	2224	0.912	792
region.rates3	0.931	563	0.783	1309	0.788	1068	0.467	806
region.rates4	0.996	608	0.813	1055	0.719	1003	0.514	648
region.rates5	0.708	636	0.827	979	0.522	1105	0.491	696
region.rates6	0.889	721	1.015	1360	0.831	1030	2.135	1579
region.rates7	1.041	611	0.979	1136	0.813	1013	1.523	903
region.rates8	0.818	552	1.356	1769	0.705	1013	0.925	883
region.rates9	2.574	1495	1.204	1469	0.496	916	0.937	895
region.rates10	0.704	681	0.913	867	0.916	1218	1.041	582
region.rates11	0.797	582	-	-	-	-	1.011	830
region.rates12	0.916	600	-	-	-	-	0.903	493
region.rates13	1.17	834	-	-	-	-	1.974	1273
region.rates14	0.645	638	-	-	-	-	0.964	740
region.rates15	0.848	639	-	-	-	-	0.87	856
region.rates16	0.714	626	-	-	-	-	-	-
region.rates17	1.101	733	-	-	-	-	-	-
region.rates18	0.91	618	-	-	-	-	-	-
region.rates19	0.808	550	-	-	-	-	-	-

region.rates20	0.725	546	-	-	-	-	-	-
region.rates21	1.704	858	-	-	-	-	-	-
treeLikelihood	-1899.088	3122	-1537.361	2821	-2378.417	2208	-1526.125	1976
region.treeLikelihood	-44.251	1919	-31.157	3294	-33.405	1635	-50.53	2147
coalescent	-112.004	1980	-83.229	1377	-113.236	1378	-149.324	1055

Figure S1 – All specimens, for which we had coordinates, plotted on a word map.

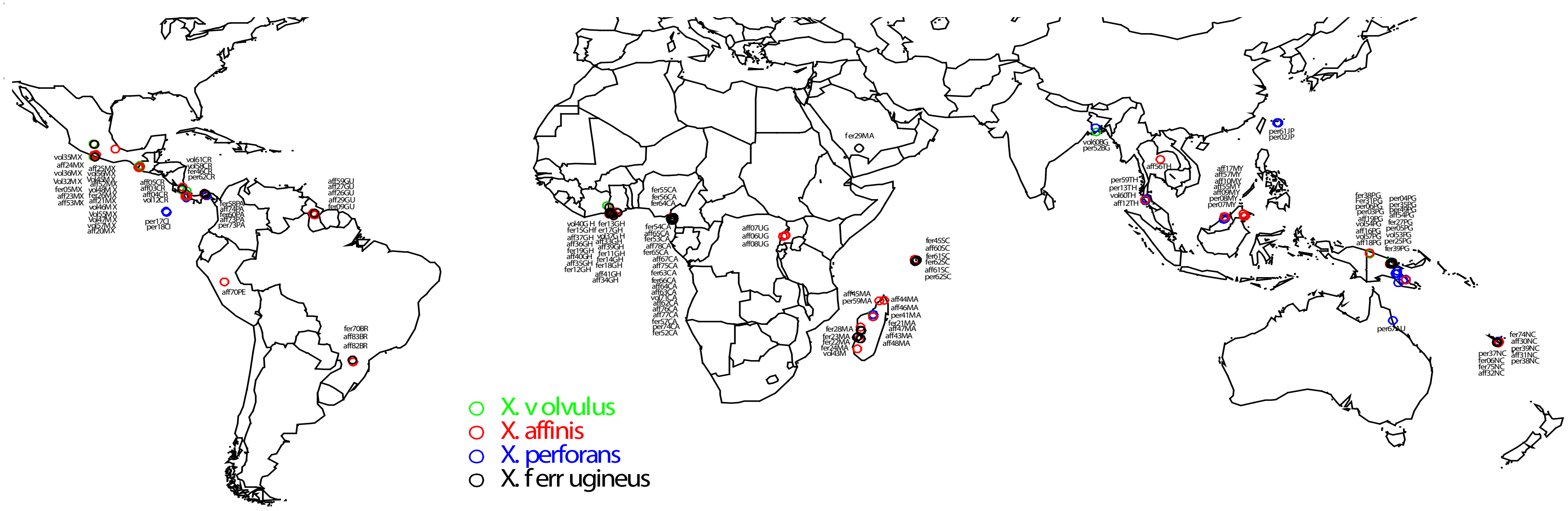

Figure S2 – A tanglegram showing the level of concordance between the two phylogenetic markers (COI and EF1α) used in the phylogenetic and biogeographic reconstruction shown in figure 1. Here we include only the individuals that were sequenced for both markers. Clades that are represented in both phylogenies are indicated by solid branches. The trees were generated by BEAST and node labels indicate posterior probability.

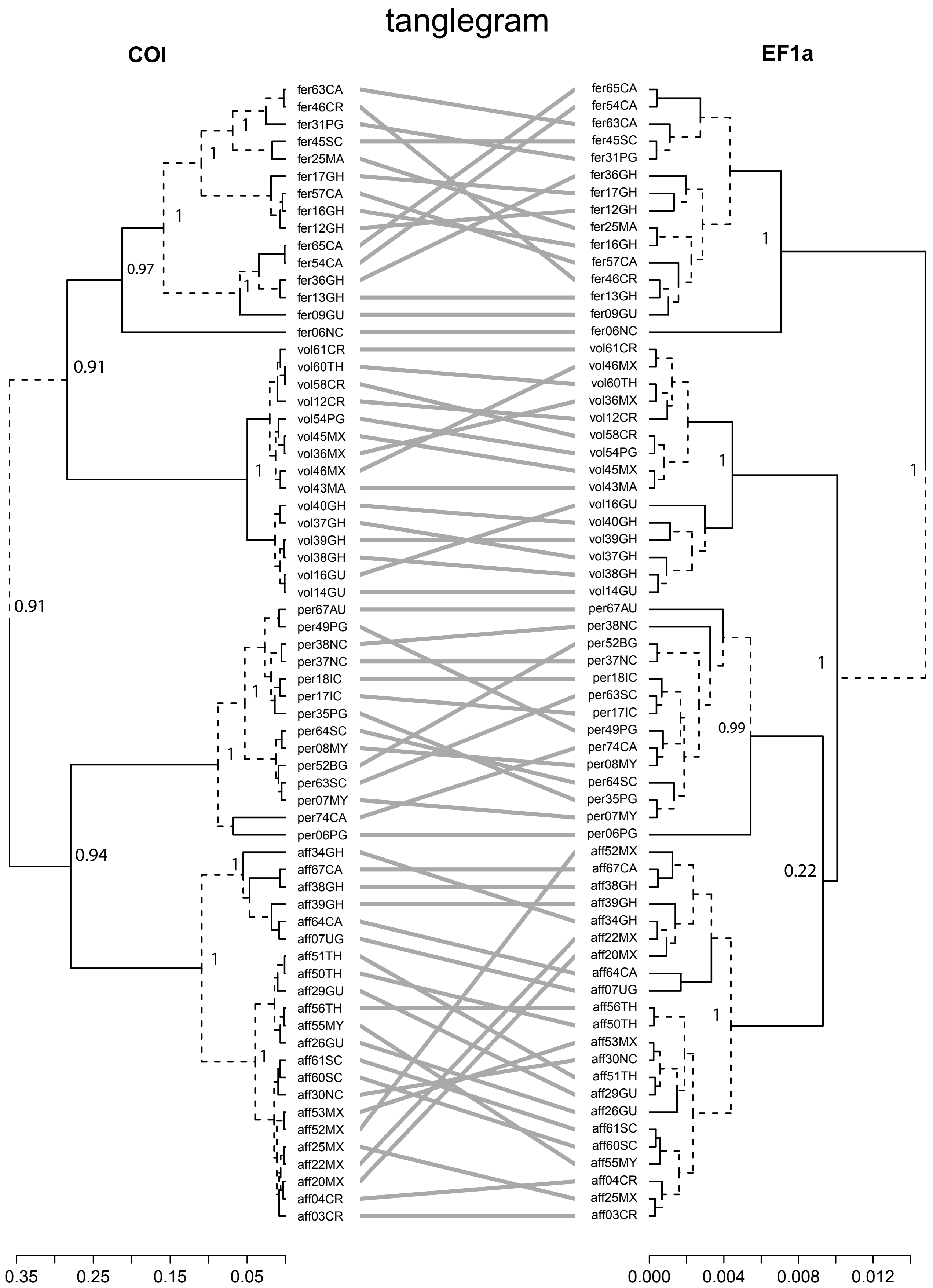

1τ

Figure S3 - Mismatch distribution plots showing the distribution of distances between alleles expected under stable population size (red line; Rogers & Harpending 1992) and the empirical data (blue line and histogram).

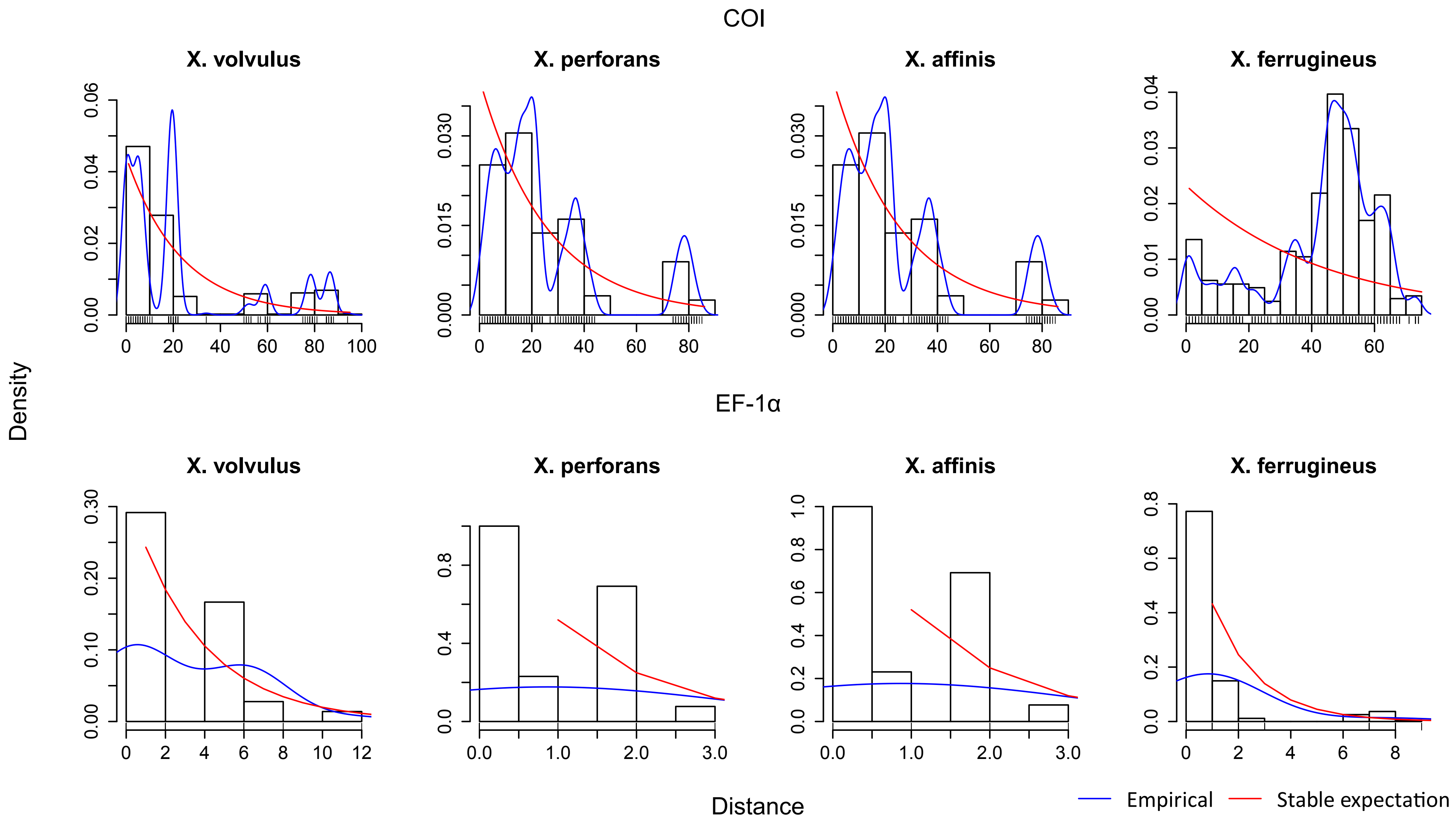

Figure S4 – EF1α haplotype network.

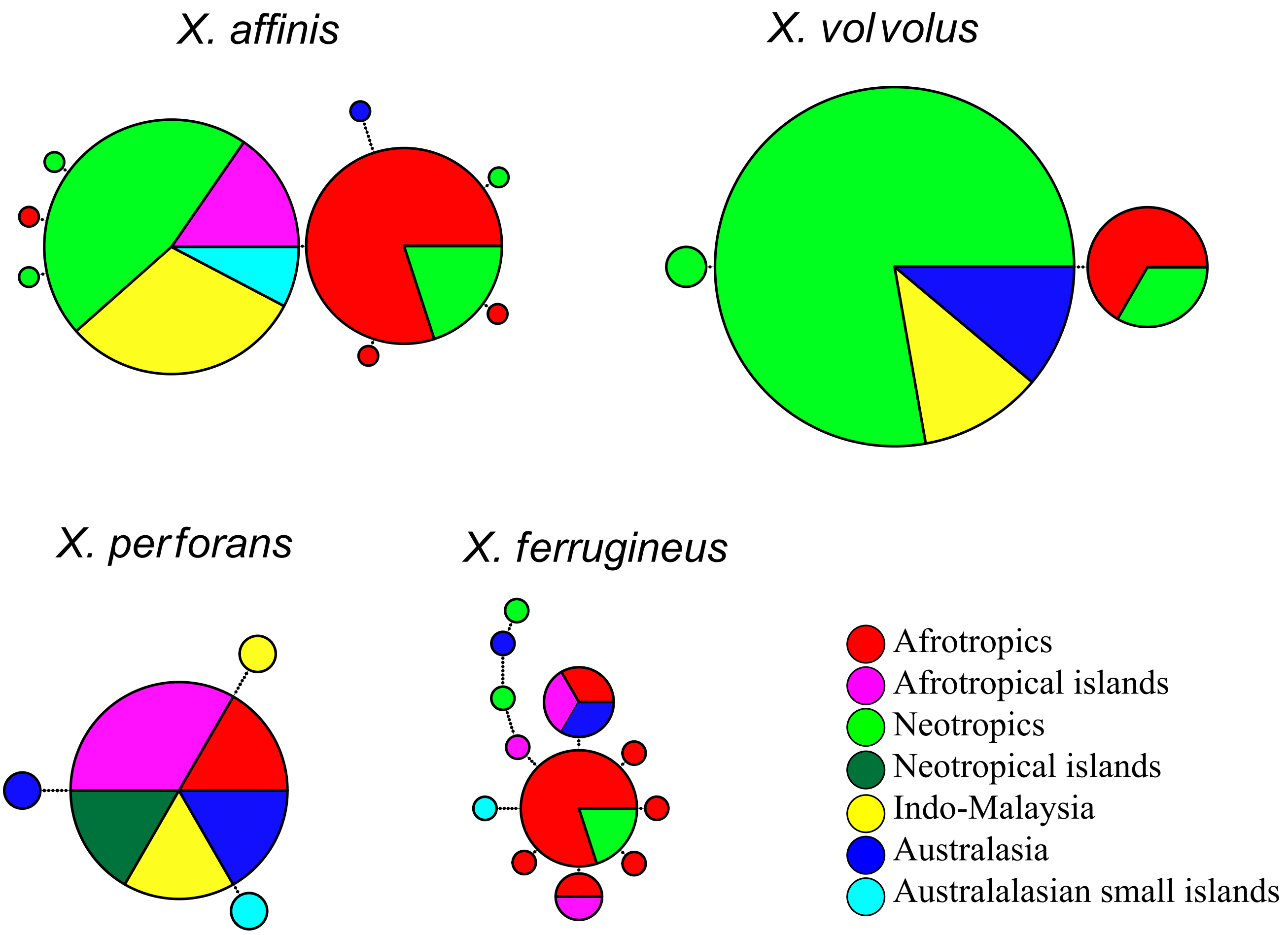

Figure S5 - Mantel tests of genetic and geographic distance.

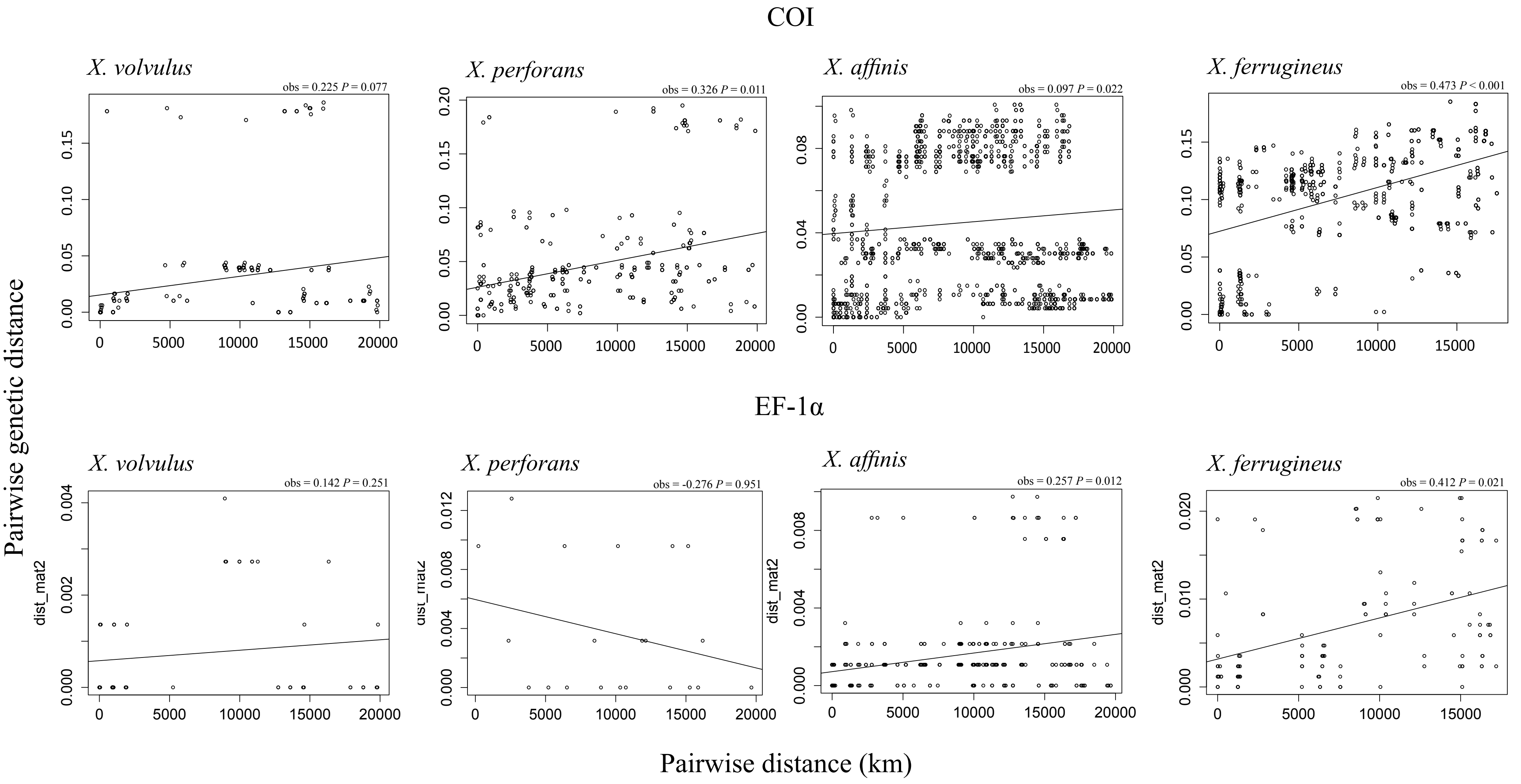

Literature

Normark BB, Jordal BH, Farrell BD (1999) Origin of a haplodiploid beetle lineage. *Proceeding of the Royal Society of London Series B.* **266**, 2253-2259.

Rogers AR, Harpending H (1992) Population growth makes waves in the distribution of pairwise genetic differences. *Molecular Biology and Evolution* **9**, 552-569.
